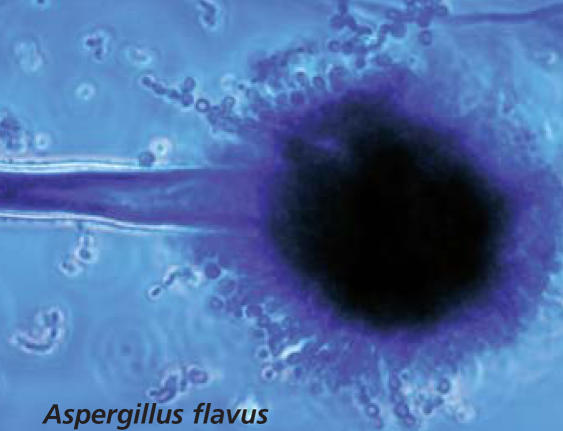# EHPnet: Doctor Fungus

**Published:** 2007-06

**Authors:** Erin E. Dooley

The Doctor Fungus website, located at **http://www.doctorfungus.org/**, strives to be an online reference source for all things mycological. The nonprofit site is continually revised, updated, and reviewed by an international team of pathologists, mycologists, pharmacologists, and other specialists. Although intended primarily for physicians, the site offers plenty of information for an educated lay audience. The site’s resources are searchable by a number of ways: through topic headings down the middle of the homepage, a toolbar at the top of the homepage, a pull-down menu at the top of the home-page, and a sitewide search engine.

The Think You Have a Fungal Infection? section of the site has information on fungal infections in people, animals, and plants, plus a separate section devoted to yeast infections. The human portion has links to information on nine common fungal infections and a section on miscellaneous infections that can be caused by more than one type of fungus. Each disease is defined and described, color images of the fungus are available, and readers can learn about prognosis, therapy, histopathology, and laboratory tests used to confirm the presence of the fungus. Other information includes an overview of the natural habitat of each fungus and susceptibility testing where relevant. Links to other sites with information on the fungus are listed along with access to PubMed for searching for further research articles on the topic.

The Think You Have a Sick Building? section offers instructions on how to look for mold in buildings, both on surfaces and in the air. Overviews of the three primary tools for air analysis are provided. Also within this section are pages on mold remediation and lists of frequently asked questions for homeowners and physicians.

The Learn About Fungus section has two overviews of mycology, one for the general public and one for those with more extensive scientific expertise. Descriptions of the most prevalent types of fungi are also available in this section. Each description contains links to images and information on susceptibility. Also featured are the taxonomic classification of the fungus selected, information on pathogenicity, clinical significance, and macro- and microscopic features. There is also a page of fungal names and synonyms.

The Antifungal Agents section addresses drugs used to treat fungal infections. The Introduction page of this section has links to the six available classes of agents used in treating such infections. A page on antifungal pharmacology discusses the fungal cell structure and targets. Additional pages within this section address nephrotoxicity, drug dosing in renal and liver dysfunction, and drug interactions.

## Figures and Tables

**Figure:**